# Sanitas Oil

**Published:** 1886-04

**Authors:** A. W. Harlan

**Affiliations:** Chicago, Ill.


### SANITAS OIL.

BY A. W. HARLAN, M. D., D. D. S., CHICAGO, ILL.

On page 19 of the INDEPENDENT PRACTITIONER for January, 1886, will be found a short article on "Sanitas," by Dr. E. S. Talbot, of Chicago, attempting to disprove a well established fact—that Sanitas is a disinfectant. He says: "I was induced to try its disinfecting qualities (properties) from the following remarks of Dr. Harlan, * * page 269, *Ohio State Journal* for June: 'Sanitas is a disinfectant. * * * Its elements are capable of combining with sulphuretted hydrogen gas and other products of decomposition, and thereby destroying or removing them.' The first quality manifested was its odor, which remained for a great length of time. I made two applications to the root of an incisor, and was unable to treat further, as the patient could not endure the taste in the mouth. (The cavity should have been sealed.) I passed a probe into the canal, and the odor of Sanitas was stronger than sulphuretted hydrogen. I suspected that it had not disinfected, but its own powerful odor had covered other odors. I consequently commenced a series of experiments, (he only relates one,) and think anyone may confirm the results if he will do likewise."

Here is the experiment:

"I put a small quantity of $H_2$ S. in a test tube and added some water (he does not say how much water), then put a drop of acetate of lead (an aqueous solution, probably), on a strip of white paper and inserted it into the tube, which immediately turned dark. I put a few drops of Sanitas into the test tube, corked and shook it well, and let it remain a few minutes. I moistened the other end of the white paper with acetate of lead (aqueous solution), and dipped it into the test tube. The dark color is the same as before, showing that no chemical change occurred by adding Sanitas."

On two or three occasions the writer has ventured to call attention to new drugs, but more frequently his efforts in this direction have been limited to pointing out new uses for drugs, when they appeared to possess more than one desirable or useful property. Such was my object in calling attention to Sanitas Oil. It may be that some of the readers of the INDEPENDENT PRACTITIONER do not know what it is.

Sanitas Oil, which is oxidized oil of turpentine, was discovered by Mr. C. T. Kingzett, F. I. C., F. C. S. "It is prepared by exposing a large quantity of turpentine floating upon water to a blast of hot air, when the turpentine is oxidized and increased in density, giving rise to the production of camphoric peroxide, camphor, and various other substances of an oxidized nature. Some of these products, notably peroxide of hydrogen, camphoric acid and thymol, dissolve in the water forming the Sanitas fluid. Floating upon the surface of the solution is the oxidized oil of turpentine, Sanitas Oil."—(Stocken).

"I now come to another and more important matter, and that is to announce the discovery of what is, I believe, a better antiseptic than oil of eucalyptus for use in surgery; at the same time it is chemically allied to that substance, and but for economical considerations, can be produced therefrom. The product to which I refer is an oxidized oil of turpentine, in other words, the product which I obtain by forcing a current of air through turpentine in the presence of water (or otherwise) during a prolonged period, lasting in practice from one to two hundred hours. By this process of air oxidation the turpentine absorbs an enormous quantity of oxygen, increasing in density accordingly, and losing its very volatile character. Although the product is not, strictly speaking, soluble in water, it forms in contact therewith, or even in contact with any moist surface such as is presented by every wound, certain principles which pass into aqueous solution. These principles are strongly antiseptic, and amongst them peroxide of hydrogen is to be numbered. * * * It is freely soluble in spirit, and is perfectly miscible with other oils, and with petroleum bases, etc. A gauze can be readily made from it which keeps its strength, so far as I can judge, very well; and what is of more importance, it is a very powerful oxidant as well as an antiseptic."—*Lancet*, June 11, 1881. C T. Kingzett, F. C. S.

"Sanitas Oil exhibits the same general properties (germicidal, antiseptic, and disinfectant), in a very intense degree. In oxidizing power it is equal to a ten volume solution of peroxide of hydrogen, while in antiseptic intensity it equals phenol, thymol and iodoform. It is altogether superior to oil of eucalyptus." Address to members of the medical profession by C. T. Kingzett, F. C. S., Vol. 1885, Sanitas.

" * * * But there is another preparation * * * and that is the Sanitas oil. This substance contains an organic peroxide, and continually yields peroxide of hydrogen to water when placed in contact therewith. Whether, therefore, the oil be used alone or in the form of an emulsion made from it and gum acacia and water, or as a mixture with olive or other oils, it steadily goes on producing peroxide of hydrogen when in contact with wounds or mucous surfaces, in which, of course, water is ever present."—Peroxide of Hydrogen, etc., *Brit. Med. Jour.* Dec. 2, 1882.

"THE OWENS COLLEGE,
MANCHESTER, England, April 24, 1881.

"C. T. KINGZETT, Esq.,

"*Sir*,—I have pleasure in hereby certifying that Sanitas oil and Sanitas powder are in every respect equal to one or other of the disinfectants now on the list of the Marine Department of the Board of Trade. Also that these two substances act partly as antiseptics, like carbolic acid, and partly as oxidants, like Condy's Fluid. Also that the Sanitas preparations contain no hurtful or poisonous ingredients. Yours, etc.,

"H. E. ROSCOE,"

(Report on Sanitas oil by Prof., T. E. Thorpe, Ph.D., F. R. S., Prof. Chemistry "The Yorkshire College, Leeds.")

1st. Sanitas oil is obtained by forcing warm air for many hours through a mixture of oil of turpentine and water in varying proportions. * * Large quantities of a camphorated body are formed, which, in contact with water, generates peroxide of hydrogen, which passes, together with small quantities of other products, into water mixed with turpentine. The oxidized oil * * * contains a considerable amount of the body, which, in the presence of water forms hydrogen-peroxide, still undecomposed. On agitating the oil with successive portions of water, the peroxide may be readily detected in the aqueous solution. There is reason to believe that the oxidized body thus formed in the turpentine is the camphoric peroxide discovered by Sir Benj. Brodie.

2d. The experiments on the behavior of Sanitas towards putrescible substances were begun during the last week in October, and have only just been brought to a close (Dec. 20th), * * * The oil has a much stronger or more persistent action than the fluid or powder.

*Experiments*—A piece of beefsteak suspended in water containing one cubic centimetre of Sanitas oil remained sweet for thirty-five days, whilst a similar piece in water containing no oil was putrid on the second day; so also was a piece of steak to which one-tenth of a cubic centimetre of Sanitas oil had been added. A solution containing one-half cubic centimetre preserved the beef four days. Flour-paste and white of egg remain apparently unchanged for much longer periods of time under the influence of Sanitas oil. Flour-paste containing one-half cubic centimetre of the oil remained perfectly sweet so long as the observations were continued (forty-seven days). Solutions containing ten cubic centimetres of white of egg, diluted with fifty cubic centimetres of water, experienced no apparent change in contact with one-half cubic centimetre of the oil during the whole period of the experiments, whilst both the flour-paste and white of egg solutions unmixed with the oil were sour and putrid within a week after being made. * * * Sanitas oil, like the fluid, is innocuous and volatile, and is not liable to deteriorate through changes of temperature or of climate. It has a strong aromatic smell, which, when diluted with air, is not unpleasant, a quality in which it differs from carbolic acid. It is difficult to say with accuracy how much more powerful it is than Sanitas fluid in its anti-putrescent action. Judging, however, from the amount of oxygen which it is capable of liberating, it cannot be less than from ten to twelve times as potent. This conclusion is roughly confirmed by observations on the rate of change of putrescible substances mixed with known amounts of the fluid and the oil."

"Sanitas oil, indeed, is the most powerful in its action, being fully as active in power of oxidation as a ten volume solution of peroxide of hydrogen. On putrid urine Sanitas was the only disinfectant that really had a true action, the others merely covering the smell by their own. I found the Sanitas oil liberated more iodine than the same quantity of ten volume solution of peroxide of hydrogen." James Baynes, Jun., F. C. S., Public Analyst for Hull, England.

Le Sanitas. Il renferme de l'eau oxygénée, bien connue pour ses effets oxydants, extrémement énergiques, quî la rendent éminement propre à la désinfection et à l'empêchement de la putréfaction, en sorte qu'elle aurait sans doute été employée depuis longtemps comme désinfectant et comme antiseptique, si son prix élevé n'avait pas été un obstacle à son emploi. En tout cas, la richesse en eau oxygénée, seule assure au Sanitas le premier rang entre les articles employés dans un but semblable.

Docteur O. BILLETER, Prof. de Chîmie.

Neufchatel, Suisse.

"When oil of turpentine is exposed to the air it slowly becomes solid, absorbing oxygen and becoming converted into resinous bodies. Among these bodies there is found a small quantity of camphoric peroxide ($C_{10} H_{14} O_4$) which undergoes decomposition in contact with water, yielding camphoric acid and hydric peroxide; ($C_{10} H_{14} O_4 + 2 H_2 O = C_{10} H_{16} O_4 + H_2 O_2$). This explains the observation that old oil of turpentine exhibits many of the reactions of hydric peroxide. By passing air and steam through oil of turpentine, a powerfully oxidizing solution containing hydric peroxide has been prepared by Kingzett, and proposed under the name of "Sanitas" for disinfecting purposes. It is worthy of remark that the leaves of the Eucalyptus Globulus, so much esteemed for its sanitary influence, also yield an oil similar to oil of turpentine, which becomes brown and resinous when exposed to air." Bloxam's Chemistry, pp. 475-6.

70 DEARBORN ST., CHICAGO, ILL., Feb. 25, 1886.

Prof. C. B. GIBSON, Ph.G.

*Dear Sir*,—Enclosed you will find two bottles labeled No. 1 and No. 2, each containing about one fluid ounce; will you be kind enough to test their disinfecting properties on $H_2 S$ and $P H_3$ and report the result to me at your earliest convenience, and greatly oblige,

Yours very truly,

A. W HARLAN.

(Reply.)

College of Physicians and Surgeons
Chicago Chemical Laboratory.

CHICAGO, 3–10, 1886.

*Dear Sir,*—I have made several experiments with the liquids you left for me. As regards their disinfecting properties when brought in contact with sulphuretted hydrogen and phosphoretted hydrogen, I used saturated aqueous solutions of these gases and made several tests in each case, using lead acetate solution and silver nitrate solution as indicators for $H_2$ S and $H_3$ P respectively. I find that 1 vol. of either the liquid or oil will easily deodorize and destroy the $H_2$ S solution in twenty-four to thirty-six hours completely, no indications of its presence being given with lead acetate test. One vol. of the oil or liquid will completely deodorize five vols. of the $H_3$ P solution in twenty-four hours, no indication of $H_3$ P remaining. The oil is much more effective than the aqueous liquid, and would undoubtedly give a much better showing with more extended experiments. These tests were, of course, many times more severe than you would subject the substances to in actual practice, as the solution contained three and one-quarter vols. of $H_2$ S to one vol. of water, and the $H_3$ P solution was fully saturated. These liquids are undoubtedly powerful disinfectants, and must prove very valuable in the practice of dental surgery.

Respectfully yours,
CHAS. B. GIBSON, Prof. Chemistry.

Prof. A. W. HARLAN, M. D., D. D. S.

The bottles above mentioned contained Sanitas oil and Sanitas fluid. While temporarily sojourning in London this winter, I wrote to Mr. Kingzett, stating that a denial had been made by Dr. Talbot of the disinfecting property of Sanitas oil. I received a reply too long to quote entire. He says: " * * * From time to time unscientific men, who are not at all qualified to write upon the subjects in question, make statements which it is scarcely worth my while to combat, and doubtless the remarks which you have seen in a late number of the INDEPENDENT PRACTITIONER, are of this character. It is positively absurd for any man who has the smallest smattering of chemistry to deny that Sanitas, containing as it does peroxide of hydrogen as one of its constituents, can decompose hydrogen sulphide, for every chemist knows that it is quite impossible for the two substances to exist in mixture, the hydrogen sulphide being immediately decomposed by the peroxide."

I think from what has already been quoted that I need not burden the readers of this journal with any more proofs of my original statement, which was that "Sanitas is a disinfectant. * * * Its elements are capable of combining with sulphuretted hydrogen gas and other products of decomposition, thereby destroying or removing them."

I will say that I did not rely on my sole experiences of the value of Sanitas when I wrote my first paper, briefly describing its properties, but I had read the commendatory remarks of Messrs. T. Charters White, M. R. C. S., L. D. S., Fred. Bate, L. D. S., Yurnell Hammond, L. D. S., W. R. Humby, L. D. S., "Nature's Hygiene, 1st edition, Baillière, Tindall & Cox, Publishers, and several other very flattering notices. In conclusion permit me to express the opinion that Sanitas is perhaps the sole substance not poisonous to human life, at the present time, which is certainly destructive of ærobic and anærobic germs (Pasteur), and I believe, also, that there are few scientific practitioners of dental surgery who can dispense with its use after having fully and carefully tested it in daily practice.

